# A multicentre retrospective cohort study on health-related quality of life after traumatic acute subdural haematoma: does cranial laterality affect long-term recovery?

**DOI:** 10.1186/s12883-022-02790-3

**Published:** 2022-08-01

**Authors:** V. D. N. Hoogslag, T. A. van Essen, M. D. Dijkman, W. Moudrous, G. G. Schoonman, W. C. Peul

**Affiliations:** 1 University Neurosurgical Centre Holland, LUMC, HMC, Haga, Albinusdreef 2, 2333 ZA Leiden &The Hague, The Netherlands; 2 Maasstadziekenhuis, Rotterdam, The Netherlands; 3 Elisabeth-TweeSteden Ziekenhuis, Tilburg, The Netherlands

**Keywords:** ASDH, Acute subdural haematoma, Acute subdural hematoma, Laterality, Quality of life, Health-related quality of life, Qolibri

## Abstract

**Background:**

Traumatic acute subdural haematoma is a debilitating condition. Laterality intuitively influences management and outcome. However, in contrast to stroke, this research area is rarely studied. The aim is to investigate whether the hemisphere location of the ASDH influences patient outcome.

**Methods:**

For this multicentre observational retrospective cohort study, patients were considered eligible when they were treated by a neurosurgeon for traumatic brain injury between 2008 and 2012, were > 16 years of age, had sustained brain injury with direct presentation to the emergency room and showed a hyperdense, crescent shaped lesion on the computed tomography scan. Patients were followed for a duration of 3-9 months post-trauma for functional outcome and 2-6 years for health-related quality of life. Main outcomes and measures included mortality, Glasgow Outcome Scale and the Quality of Life after Brain Injury score. The hypothesis was formulated after data collection.

**Results:**

Of the 187 patients included, 90 had a left-sided ASDH and 97 had a right-sided haematoma. Both groups were comparable at baseline and with respect to the executed treatment. Furthermore, both groups showed no significant difference in mortality and Glasgow Outcome Scale score. Health-related quality of life, assessed 59 months (IQR 43-66) post-injury, was higher for patients with a right-sided haematoma (Quality of Life after Brain Injury score: 80 vs 61, *P =* 0.07).

**Conclusions:**

This study suggests patients with a right-sided acute subdural haematoma have a better long-term health-related quality of life compared to patients with a left-sided acute subdural haematoma.

**Supplementary Information:**

The online version contains supplementary material available at 10.1186/s12883-022-02790-3.

## Background

Traumatic brain injury (TBI) is rising in incidence and is a major cause of morbidity and mortality. Those who survive often suffer from lifelong disability [1, 2]. It is estimated that approximately 5.3 million people in the USA and approximately 7.7 million people in the European Union are living with disabilities related to TBI [3, 4]. Acute subdural haematoma (ASDH) is one of the most harmful forms of TBI [5–7]. The haemorrhage is located in the subdural space and is often associated with a contusion or an intracerebral haematoma [3, 6]. A traumatic ASDH can be treated either surgically or conservatively. Surgical treatment may consist of evacuation of the haematoma and can be complemented by a decompressive craniectomy (DC) (i.e. leaving the bone flap out) to lower the intracranial pressure (ICP). In some cases surgery is not needed and patients recover with surgically expectant management in intensive or medium care units [7]. There is no consensus on optimal management strategy [8, 9]. The complexity of this decision lies in the balance between liberal surgical indications with an increased number of survivors with an anticipated unacceptable health-related quality of life (Hr-QoL) against conservative (surgically expectant) management with unnecessary death and disability.

Laterality of the haematoma does not play a major role in the decision for surgery, as is reflected in the absence of consideration in TBI guidelines [10]. Furthermore, only a limited amount of research regarding this laterality is available [11–14]. This contrasts with stroke laterality where numerous studies have shown an association of worse outcome in dominant hemispheric infarction [15–17]. It is possible the same distinction in influence of location on outcome pertains to ASDH patients. Therefore, the aim of this study is to investigate the influence of the laterality of the ASDH on the recovery of the patient regarding mortality, functional outcome and Hr-QoL.

## Method

### Study cohort and eligibility criteria

This is a retrospective observational cohort study of all ASDH patients treated consecutively between 2008 and 2012 by three Dutch neurosurgical departments in level 1 trauma centres [18]. The two regions in which these centres are located had a homogeneous population of around 2 million each. All patients were retrospectively identified using the treatment codes of the Dutch registry system for hospital funding. To limit the amount of missed inclusions, the national trauma registry was checked [19]. The data was independently gathered according to a standardized collection sheet and all data was subsequently collected by two physicians employed in the same region.

Patients were included if they were older than 16 years, had a clinical diagnosis of blunt traumatic brain injury, presented directly to the emergency room and showed a hyperdense, crescent shaped lesion – indicative of an ASDH – on the computed tomography (CT) scan. Exclusion criteria were non-traumatic ASDH, ASDH secondary to an earlier procedure, penetrating injury and concomitant focal intracranial lesions (i.e., intracerebral haematoma or epidural haematoma) that required emergency surgery. Patients who were withheld from treatment because they were deemed unsalvageable or had severe comorbidity, were excluded as well.

The study was approved by the medical ethics committee of the Leiden University Medical Centre. All patients and caregivers who participated gave written informed consent.

### Variables

Data on demographics, medical history, use of anticoagulants or antiplatelet agents, injury-related variables, radiological variables, treatment variables, complications, and outcome variables were collected from electronic patient files.

Injury-related variables included trauma mechanism, first emergency room Glasgow Coma Scale (GCS) score, the severity of the patients’ initial GCS score (GCS 13-15: Mild, GCS 9-12: Moderate, GCS 3-8: Severe), focal neurological symptoms (paresis, aphasia, or cranial nerve deficit), pupillary light reflex, clinical deterioration (i.e., a decrease of > 1 point on the GCS, new abnormal pupillary light reflex, or new focal neurological symptoms, from the time of first assessment).

The first CT scan was used to assess the radiological variables that included the thickness of the haematoma, the amount of midline shift (MLS), the presence of concomitant contusion and the patency of the basal cisterns. Furthermore, if a second preoperative CT scan was made, it was noted whether it showed radiological deterioration (i.e. > 5 mm increase in haematoma thickness or presence of new focal lesion).

Therapy-related variables consisted of the maximum ICP and the type of management (conservative or surgical). The treatment was considered conservative when the neurosurgeon on call decided not to operate acutely. It was considered surgically when a report was made on the indication and the surgical procedure was started after the last CT ordered or performed in the emergency room. Decompressive craniectomy was defined as such when the neurosurgeon did not reconstruct the bony defect after the craniotomy.

Furthermore, all complications during admission that required medical attention (e.g. antibiotic treatment for infection) were noted. Complications were defined as intracranial (e.g. seizure, hydrocephalus), cardiovascular (e.g. arrhythmia, ischemia), respiratory (respiratory insufficiency, hypoxia), metabolic (e.g. electrolyte disturbances, renal failure), infections (e.g. pneumonia, wound infection), or other.

Outcome measures were mortality at discharge, functional outcome and the Hr-QoL. Hr-QoL was the primary outcome measurement for this study. Functional outcome was judged according to the 5-point Glasgow Outcome Score (GOS) (dead, vegetative, severe disability, moderate disability, good recovery) derived from outpatient follow-up letters at three to 9 months post-trauma which were dichotomized into GOS favourable (GOS 4–5) and unfavourable (GOS 1–3). The Hr-QoL was assessed using the TBI-specific Qolibri questionnaire [18, 20–23]. It was determined at least 2 years after the injury to ensure a long-term overview of outcome. The Qolibri is a comprehensive 37-item questionnaire investigating six dimensions that are typically affected after TBI. Patients rate their (dis)satisfaction (1-5 scale) on six subscales representing the dimensions: cognition, self, daily life & autonomy, social relationships, emotions and physical problems. Scores are transformed to total scores ranging from 0 (worst possible Qolibri) to 100 (best possible Qolibri). Patients were asked to complete and return the questionnaire by post. In case patients did not return the questionnaire, the investigators attempted a telephone interview, or family members were asked to assist in completing the forms. In addition, the reason for not returning the questionnaire (death or persistent unresponsive state) was collected [23].

### Statistical analysis

The statistical analysis was done using IBM SPSS Statistics 23. To assess differences between groups, appropriate tests were used according to distribution and scale of measurement.

(Student’s t-tests or Man-Whitney U test for the continues values and a Chi-square test for the categorical values) and reported in *p*-values and effect size r. A difference was considered significant with a *p* < 0.05. In order to provide a summary baseline prognostic score for both groups, a multivariable logistic regression model, based on all available variables featured by Corticosteroid Randomization After Significant Head Injury (CRASH) CT head injury prognosis model (age, GCS score, pupil reactivity to light, major extracranial injury, midline shift > 5 mm, traumatic subarachnoid haemorrhage, and obliteration of the basal cisterns), was used to estimate a calculated predicted probabilities for mortality and unfavourable outcome [24]. In sensitivity analysis, the analysis was restricted to those patients with ASDH that do not have a contusion.

## Results

**Fig. 1:**
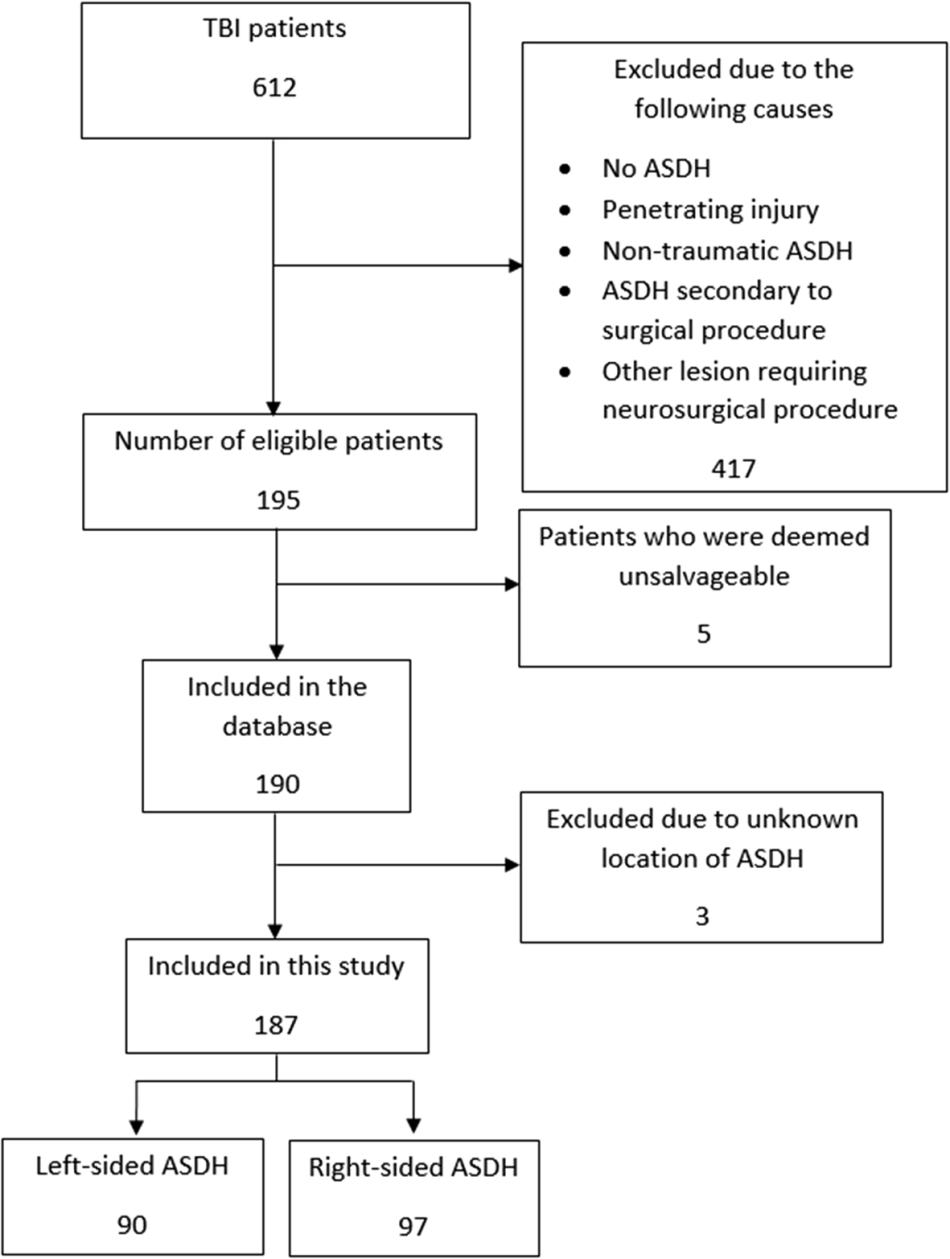
Patient flow chart

Out of 612 screened consecutively admitted patients, 190 were included. For this study three patients were excluded due to unknown location of the ASDH. Of the remaining 187 patients, 90 had a left-sided ASDH and 97 had a right-sided ASDH (Fig. 1).

Both ASDH groups had similar baseline characteristics. The left-sided ASDH cohort consisted of 40 females (44.4%) with a median age of 68 years (Interquartile range (IQR): 53-77). The right-sided ASDH cohort consisted of 46 patients (47.4%) were female with a median age of 68 years (IQR: 55-76). Furthermore, both groups showed no significant difference in TBI severity. Finally, the equal CRASH scores confirmed the short term (predicted mortality) and long term (predicted unfavourable outcome) baseline comparability of both groups. There were no differences in proportions undergoing surgical treatment and DC or the proportion complications (Table 1). Furthermore, with respect to clinical or radiological deterioration, the hospital course was similar (Table 2).

**Table 1:** Baseline characteristics

	ASDH left	ASDH right	***P*** value
**Sex N (%)**			0.68
Male	50 (56)	51 (53)	
Female	40 (44)	46 (47)	
Missing	0 (0)	0 (0)	
**Age at time of trauma (years)**			0.84
Median	68	68	
IQR.	53-77	55-76	
Missing (%)	0	0	
**Diabetes mellitus N (%)**			0.27
No	74 (82)	86 (89)	
Yes	14 (16)	10 (10)	
Missing	2 (2)	1 (1)	
**Cardiovascular disease N (%)**			0.61
No	37 (41)	44 (45)	
Yes	51 (57)	52 (54)	
Missing	2 (2)	1 (1)	
**Use of anti-coagulants or thrombocytes inhibitors N (%)**			0.72
No	50 (56)	54 (56)	
Anti-coagulants	20 (22)	25 (26)	
Thrombocyte’s inhibitors	17 (19)	13 (13)	
Both	3 (3)	2 (2)	
Missing	0 (0)	3 (3)	
**Focal neurological symptoms N (%)**			0.73
No	35 (39)	36 (37)	
Yes	32 (36)	37 (38)	
Missing	23 (26)	24 (25)	
**Pupillary light reflex N (%)**			0.79
No	66 (73)	63 (65)	
One absent	11 (12)	14 (14)	
Two absent	12 (13)	11 (11)	
Missing	1 (1)	9 (9)	
**Initial GCS**			0.24
Median	9	11	
IQR	6-13	6-15	
Missing (%)	4	1	
**M score**			0.13
Median	5	6	
IQR	4-6	5-6	
Missing (%)	32	28	
**Severity GCS N (%)**			0.54
Mild	28 (31)	38 (39)	
Moderate	19 (21)	19 (20)	
Severe	41 (46)	39 (40)	
Missing	2 (2)	1 (1)	
**Thickness of ASDH (mm)**			0.57
Median	13	12	
IQR	8-17	8-16	
Missing (%)	4	7	
**MLS (mm)**			0.45
Median	11	10	
N	86	93	
IQR	5-16	5-14	
Missing (%)	4	4	
**Other lesion N (%)**			0.43
No	45 (50)	44 (45)	
Yes	42 (47)	52 (54)	
Missing	3 (3)	1 (1)	
**Concomitant contusion N (%)**			0.80
No	64 (71)	73 (75)	
Yes	22 (24)	23 (24)	
Missing	4 (4)	1 (1)	
**Treatment N (%)**			0.27
Conservative	18 (20)	26 (27)	
Surgery	72 (80)	71 (73)	
Missing	0 (0)	0 (0)	
**Primary surgical treatment N (%)**			0.39
Craniotomy	39 (43)	44 (45)	
Decompressive craniectomy	32 (36)	26 (27)	
Burrhole	1 (1)	1 (1)	
Missing	0 (0)	0 (0)	
**Predicted unfavourable outcome based on CRASH-CT variables (%)**			0.89
Median	55	56	
IQR	41-75	41-73	
**Predicted mortality based on CRASH-CT variables (%)**			0.65
Median	37	30	
IQR	17-56	19-55	

*GCS* Glasgow Coma Scale score (3-15), *M score* Best Motor response score (1-6), *ASDH* Acute Subdural Haematoma, *MLS* Midline shift, *CRASH* Corticosteroid Randomization After Significant Head Injury

**Table 2:** Hospital course and outcome

	ASDH left	ASDH right	***P*** value
**Clinical deterioration N (%)**			0.75
No	46 (51)	52 (54)	
Yes	38 (42)	38 (39)	
Improvement	5 (6)	4 (4)	
Missing	1 (1)	3 (3)	
**Second CT Yes/no N (%)**			0.98
Yes	19 (21)	20 (21)	
No	74 (79)	74 (76)	
Missing	0 (0)	3 (3)	
**Second CT thickness (mm)**			0.87
Median	11	11	
N	19	20	
IQR	9-15	8-14	
Missing (%)	0	0	
**Second CT MLS (mm)**			0.58
Median	12	10	
N	19	19	
IQR	8-16	8-13	
Missing (%)	0	5	
**Second CT Cisterns N (%)**			0.46
Open	10 (11)	11 (11)	
Obliterated	9 (10)	6 (6)	
Missing	0	15	
**Radiological deterioration N (%)**			0.51
No	7 (8)	9 (9)	
Yes	12 (13)	10 (10)	
Missing	0	5	
**ICP max (mmHg)**			0.49
Median	38	30	
N	7	9	
IQR	11-70	13-53	
Missing (%)	92	91	
**Any complication N (%)**			0.65
No	32 (36)	37 (38)	
Yes	52 (58)	52 (54)	
Missing	8 (7)	8 (8)	
**Intracranial**			0.16
No	52 (58)	64 (66)	
Yes	32 (36)	25 (26)	
Missing	6 (7)	8 (8)	
**Cardiovascular**			0.40
No	72 (80)	72 (74)	
Yes	12 (13)	17 (18)	
Missing	6 (7)	8 (8)	
**Respiratory**			0.09
No	81 (90)	80 (83)	
Yes	3 (3)	9 (9)	
Missing	6 (7)	8 (8)	
**Metabolic**			0.62
No	79 (88)	82 (85)	
Yes	5 (6)	7 (7)	
Missing	6 (7)	8 (8)	
**Infection**			0.43
No	63 (70)	62 (64)	
Yes	21 (23)	27 (28)	
Missing	6 (7)	8 (8)	
**Other**			0.55
No	82 (91)	88 (91)	
Yes	3 (3)	5 (5)	
Missing	5 (6)	4 (4)	
**Mortality at discharge N (%)**			0.70
No	46 (51)	49 (51)	
Yes	35 (39)	42 (43)	
Missing	9 (10)	6 (6)	
**GOS dichotomized N (%)**			0.81
Unfavourable	49 (54)	54 (56)	
Favourable	38 (42)	39 (40)	
Missing	3 (3)	4 (4)	
**Qolibri score**			0.07
Median	61	80	
N	25	20	
IQR	52-74	56-94	
Missing (%)	38	47	
**Cognition**			0.04
Median	61	75	
N	21	19	
IQR	48 - 73	64 – 93	
**Self**			0.18
Median	61	77	
N	21	18	
IQR	38 - 70	40 – 90	
**Daily life & autonomy**			0.17
Median	50	82	
N	20	19	
IQR	28 – 69	46 – 100	
**Social relationships**			0.23
Median	63	75	
N	21	19	
IQR	48 – 81	54 – 96	
**Emotions**			0.77
Median	75	80	
N	21	19	
IQR	60 – 95	55 – 100	
**Physical problems**			0.71
Median	65	70	
N	21	19	
IQR	53 – 83	50 – 90	

CT thickness – Computed Tomography regarding thickness of the ASDH, CT MLS – Computed Tomography regarding Midline Shift, CT cisterns - Computed Tomography regarding the basal cisterns, ICP max – maximum measured Intracranial Pressure, GOS – Glasgow Outcome Scale, Qolibri score – Health-related Quality of Life after Brain Injury score

**Fig. 2:**
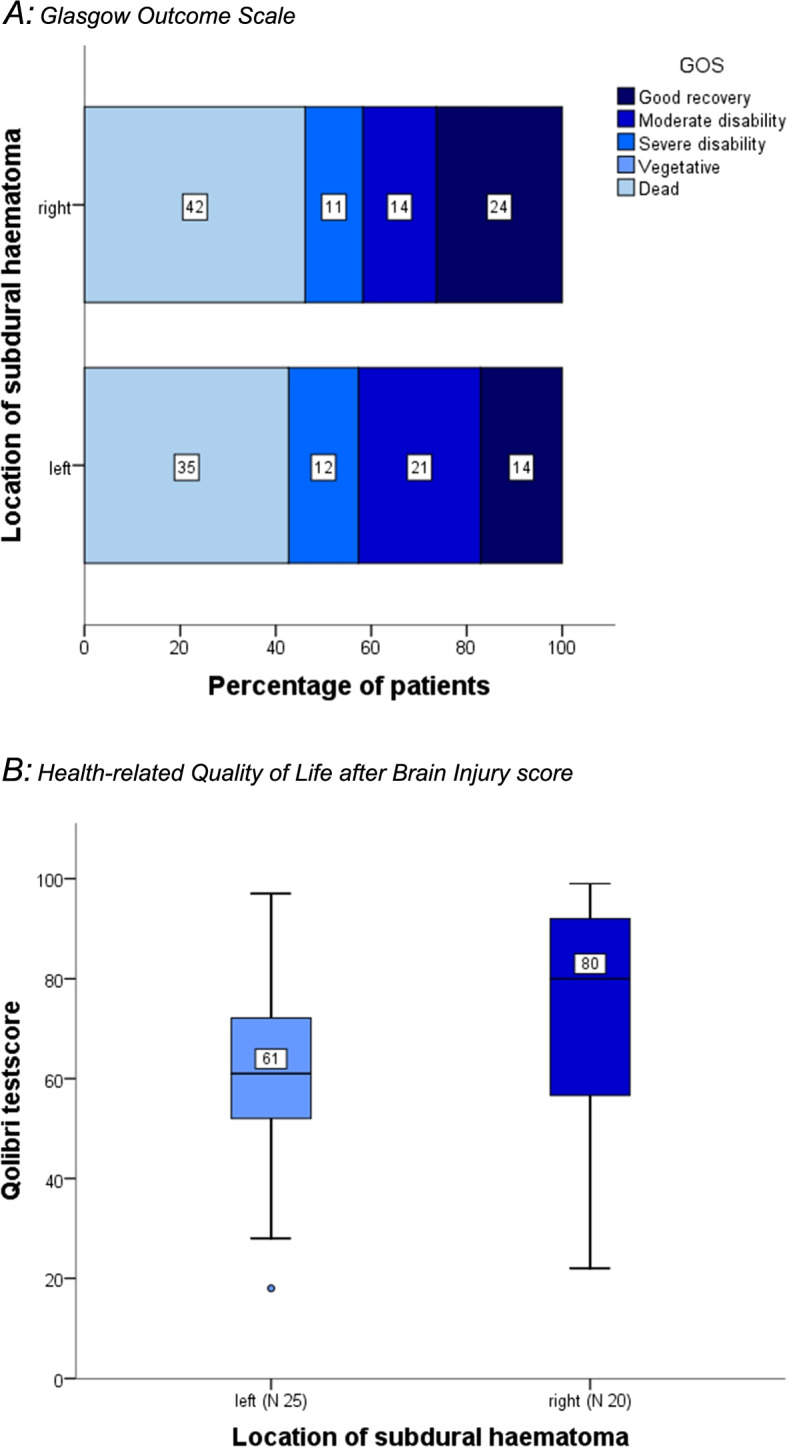
Functional outcome and Health-related Quality of Life. **A** Glasgow Outcome Scale. The Glasgow Outcome Scale (GOS) scores range from 1 (indicating dead) to 5 (Good recovery). Each cell in this graph corresponds with a GOS; the width of the cell indicates the proportion of patients with equivalent scores, and the numbers within the cell indicate the number of patients per category. **B** Health-related Quality of Life after Brain Injury score. The Quality of Life after Brain Injury score depicts the difference in health-related quality of life between left- and right sided ASDH patients. The Qolibri score is a score to indicate health-related quality of life after traumatic brain injury and is reported on a 0-100 scale (0 = worst possible health-related quality of life, 100 = best possible health-related quality of life). Each point represents a case in the respective groups. In both groups the median is represented by the numbers accompanying the points. Furthermore in this graph, both the highest and the lowest recorded Qolibri score is represented, with the highest score being 99 and the lowest score being 18

Seventy-seven patients (45%) died during hospital admission and 103 patients had a GOS < 4. The mortality and GOS did not differ between left- and right-sided haematoma patients. Subsequently, 24 patients (24%) died during the follow-up period (left, right; 11 (12%), 13 (13%)) and 8 (4%) were still severely disabled (left, right; 4 (4%), 4 (4%)), leaving 78 patients for the Qolibri assessment. Thirty-three did not respond, amounting to a loss to follow-up of 42%. The median duration of follow-up was 59 months (IQR: 43-66). Left-sided ASDH patients had a median Qolibri score of 61 (IQR: 52-74) whereas right-sided ASDH patients had a median Qolibri score of 80 (IQR: 56-94) (Table 2, Fig. 2B). The most notable result of the subscale analysis was the significant difference in cognition, suggesting a better outcome for patients with a right-sided ASDH (left 61 [IQR: 48-73] vs right 75 [IQR: 64-93], *p* = 0.04, r = 0.32). The subgroup of patients without concomitant contusion consisted of 33 patients (right *n* = 17, left *n* = 16). In sensitivity analysis excluding these patients the Qolibri scores did not differ between left and right-sided ASDH (left 62 [IQR: 53-74] vs right 83 [IQR: 47-96], *p* = 0.24).

## Discussion

In this retrospective study patients with a right-sided ASDH compared to left-sided ASDH tended to have a better Hr-QoL while having similar mortality and functional outcome. Right-sided haematoma is associated with a Qolibri score comparable with healthy individuals whereas the Qolibri score of patients with a left-sided haematoma indicate some form of persistent impairment [25]. Both groups were similar at baseline and surgically treated equally often.

To our knowledge, this is the first study to investigate the influence of laterality on functional outcome and Hr-QoL in patients with ASDH. The only previous study on hemispheric outcome difference in ASDH found a higher frequency of intractable brain swelling and mortality rate in patients with a left-sided ASDH. This difference was only significant if patients presented with a concomitant contusion [11], similar to our findings. Other studies found significant asymmetry of the autoregulatory index between the injured and intact hemisphere in TBI patients. This asymmetry in autoregulation is significantly associated with a fatal outcome, more strongly so than with a globally altered autoregulation. However, left-sided and right-sided ASDH seemed equally prone to develop autoregulatory impairment [12–14]. No other studies on the impact of ASDH laterality are available. Current guidelines for (surgical) management of ASDH do not discern between left-sided and right-sided haematomas, as evidence is lacking [10, 26, 27].

In stroke, the impact of laterality on outcome and treatment effect has been investigated elaborately. Left-sided strokes seem to fare worse considering overall outcome and seem to have a two-fold increased chance of a good outcome after thrombolysis compared to patients with right-sided hemispheric stroke [28]. Patients with a dominant sided malignant middle cerebral artery infarction are less often treated with DC. The majority of neurosurgeons expect a non-acceptable low Hr-QoL after dominant-side surgery which is not confirmed by evidence [29, 30].

The lower Hr-QoL in left-sided ASDH may be explained by the dominance of the left hemisphere and the linguistic centres. A TBI or stroke causing damage in these regions would cause dysphasia or aphasia and therefore have a more pronounced impact on Hr-QoL [31, 32]. Another possible explanation of the observed difference in Hr-QoL is that patients with an ASDH in the non-dominant hemisphere have a different experience of their Hr-QoL due to the impaired function of the right hemisphere. Cerebral focal ischemia seems to be the common pathophysiologic explanation that could explain the exceptional poor outcome of ASDH, which can be caused by generalized pressure effects through raised ICP, focal pressure effects from the haematoma, and also local toxic effects, especially in combination with ICH [33–40]. Consequently, the therapeutic mechanism of surgery could extend beyond the effect of decompressing the brain by reducing the ICP, and also serve to prevent ischemic inducing local neurotoxic events that worsen the outcome, potentially explaining the impairment of the Hr-Qol.

Research supports the notion that the perception and expression of emotion, as well as the autonomic arousal processes and awareness, are primarily represented in the right cerebral hemisphere. Patients who suffer damage of the right hemisphere often display aprosodia, asomatognosia, anosognosia, neglect, anosodiaphoria and have an impaired ability to recognise and express emotions [41, 42]. Patients with damage to the right hemisphere can present with emotional disorders which can be grouped into three sets of emotional abnormalities. Of these sets ‘emotional indifference’ is the most important one, followed by ‘verbal disinhibition’ and ‘denial of illness’ [43, 44]. This denial may cause patients to ignore the - sometimes major - handicaps or disabilities observed by healthcare professionals, causing a preserved Hr-QoL. Presumably, patients with damage to the dominant hemisphere, in contrast to patients with damage to the non-dominant hemisphere, retain a better disease insight and are less likely to ignore the functional deficits, causing a lower experienced Hr-QoL [45].

The results of this study can be applied to the majority ASDH patients presenting to a neurologist or neurosurgeon. However, some important study characteristics influence generalizability. The large proportion Dutch elderly reflects the rising age in TBI in developed countries. Furthermore, the applicability can differ between countries, since the Qolibri scores in healthy individuals differs.

This study has numerous limitations of which some are mentioned here. The small sample and retrospective design hampers definite conclusions. Furthermore, laterality is used as a surrogate for the dominant hemisphere. Another limitation is the higher loss to follow-up for left-sided ASDH which may cause selection or attrition bias. However, the proportions of patients not able to respond to the questionnaire due to death or disability was comparable in both groups. Strengths are the baseline comparability of both groups and the long-term determination of Hr-QoL at 5 years.

Future studies should re-estimate the association between ASDH laterality and outcome prospectively, while at the same time focus on the potential clinical relevance in terms of the changes over time. Long-term follow up of stroke patients has identified a possible mismatch between patients’ needs and delivered health care. Prolonged attention to healthcare needs may improve experienced Hr-QoL [46]. In this study, ASDH patients with a left-sided haematoma appeared to have a lower Hr-QoL after 5 years. In this period patients go through a medical rehabilitation process before returning home. Once back home it is possible patients and their proxy’s encounter unmet needs [46], causing a decrease in their experienced Hr-QoL. Some of these unmet needs could derive from the inability of the left hemisphere to serve as the brains’ main portal for expression of thoughts and emotions [47]. Therefore, the cognitive inabilities underlying a lower Hr-QoL in left-sided ASDH, the changes in Hr-QoL over time and, finally, the potential subsequent (rehabilitation) interventions should be studied.

## Conclusion

Traumatic ASDH is a debilitating condition with, in contrast to stroke, no known distinction in outcome between left-sided and right-sided ASDH. This multicentre observational study showed that patients with a right-sided ASDH have a higher Qolibri score compared to patients with a left-sided ASDH. This article is the first report on the existence of a difference in Hr-QoL based on location of the ASDH. Understanding the origins of this difference could provide additional insight on the laterality of brain functions, the extent to which subdural haemorrhage induces focal brain damage and ultimately prevention of the reduced Hr-QoL in patients with a left-sided ASDH.

## Supplementary Information

**Additional file 1.**

## Data Availability

The data that support the findings of this study are available from the authors but restrictions apply to the availability of these data, which were used under license for the current study, and so are not publicly available. Data are however available from the authors upon reasonable request and with permission of the authors.

## Abbreviations

ASDH: Acute Subdural Haematoma
CRASH-CT: Corticosteroid Randomization After Significant Head Injury CT
CT: Computed Tomography
DC: Decompressive Craniectomy
GCS: Glasgow Coma Scale
GOS: Glasgow Outcome Scale
Hr-QoL: Health-related Quality of Life
ICP: Intracranial Pressure
M score: Best Motor response score
MLS: Midline Shift
TBI: Traumatic Brain Injury
USA: United States of America
