# Supplementary material for: A multicentre retrospective cohort study on health-related quality of life after traumatic acute subdural haematoma: does cranial laterality affect long-term recovery?
Source: BMC Neurol. 2022 Aug 1;22:287. doi: 10.1186/s12883-022-02790-3 (PMC9341107; doi:10.1186/s12883-022-02790-3)
Supplement: Supplementary file 1 — Additional file 1. [file 12883_2022_2790_MOESM1_ESM.docx]

**Appendix 1**

**Long term recovery after excluding concomitant contusion**

|  |  | **ASDH left** | **ASDH right** | **P value** |
| --- | --- | --- | --- | --- |
| **Qolibri score** | Median  N IQR Missing (%) | 62  16  53 – 74  51 | 83  17  47 – 96  57 | 0.24 |
| **Cognition** | Median  N IQR | 64  12  48 – 76 | 75  16  46 – 98 | 0.18 |
| **Self** | Median  N IQR | 63  12  46 – 71 | 79  15  32 – 93 | 0.37 |
| **Daily life & autonomy** | Median  N IQR | 52  12  34 – 69 | 77  16  22 – 99 | 0.38 |
| **Social**  **relationships** | Median  N IQR | 71  12  50 – 91 | 75  16  54 – 98 | 0.71 |
| **Emotions** | Median  N IQR | 78  12  60 – 95 | 73  16  51 – 100 | 0.80 |
| **Physical**  **problems** | Median  N IQR | 63  12  55 – 86 | 78  16  51 – 94 | 0.67 |

Qolibri score – Health related Quality of Life after Brain Injury score
